# Evaluation of potentially inappropriate medications for the elderly according to beers, STOPP, START, and Chinese criteria

**DOI:** 10.3389/fphar.2023.1265463

**Published:** 2024-01-03

**Authors:** Xiaojuan Zhu, Feng Zhang, Yong Zhao, Wen Zhang, Yahui Zhang, Jianchun Wang

**Affiliations:** ^1^ Department of Geriatrics, Shandong Provincial Hospital Affiliated to Shandong First Medical University, Jinan, China; ^2^ Department of Clinical Pharmacy, Shandong Provincial Hospital Affiliated to Shandong First Medical University, Jinan, China

**Keywords:** potentially inappropriate medications, beers criteria, Chinese criteria, elderly, STOPP START criteria

## Abstract

**Objective:** Polypharmacy prevalence is increasing worldwide, and it is becoming more popular among the elderly. This study aimed to compare the prevalence of potentially inappropriate medications (PIMs) using the Beers criteria (2019 edition), criteria for potentially inappropriate medications for older adults in China (Chinese criteria), Screening Tool of Older Persons’ Prescriptions (STOPP), and Screening Tool to Alert to Right Treatment (START) criteria and to identify risk factors associated with PIM use.

**Methods:** This was a cross-sectional study with a sample of 276 inpatients aged ≥65 years old from January 2020 to June 2020. A cross-sectional study was conducted to analyze PIMs based on the Beers (2019 edition), Chinese, STOPP, and START criteria. PIMs use was analysed based on four different criteria and logistic regression analysis was used to investigate independent factors associated with PIM use.

**Results:** The mean number of medications used by the elderly population was nine (range, 0–28). A total of 252 patients (accounting for 91.30%) took five or more medications and 120 patients (accounting for 43.48%) took 10 or more medications. The prevalence rates of PIMs were 66.30% (183/276), 55.07% (152/276), 26.45% (73/276), and 64.13% (177/276) determined by the Beers, Chinese, STOPP, and START criteria, respectively. The top PIMs screened using the Beers, Chinese, and STOPP criteria were proton pump inhibitors, clopidogrel, and benzodiazepines, respectively. Missed use of ACEI in patients with systolic heart failure and/or coronary artery disease was found to be the most common potential prescription omission (PPOs) analyzed using the START criteria. Logistic regression analysis showed that the strongest predictor of PIMs, as determined by all four criteria, was an increased number of medications (*p* < 0.001). Age was another risk factor for PIMs based on the STOPP criteria in our study (*p* < 0.05).

**Conclusion:** Polypharmacy and PIMs were common in our study, and the risk of PIMs correlated with polypharmacy. Application of the Beers, Chinese, STOPP, and START criteria is a useful tool for detecting PIM use.

## 1 Introduction

With the increase in age and combined diseases, the use of multiple medications by one individual, termed polypharmacy, is becoming increasingly common in the elderly. Although there is no standard definition, polypharmacy is often defined as the routine use of five or more medications (Pazan and Wehling, 2021), including over-the-counter, prescription, and/or traditional and complementary medicines used by a patient. Hyperpolypharmacy or excessive polypharmacy is defined as the intake of 10 or more medications (Masnoon et al., 2017; Guillot et al., 2020; Stuhec et al., 2021; Stuhec and Zorjan, 2022).

The prevalence of polypharmacy greatly varies depending on age group, healthcare setting, and region. In a cross-sectional analysis across 17 European countries, including Israel, the prevalence of polypharmacy ranged from 26.3% to 39.9% in elderly people aged ≥65 years in the community (Midão et al., 2018). Meanwhile in a retrospective, cross-sectional study including 1,200 inpatients aged ≥65 years in the geriatric department of the hospital, polypharmacy was found in as many as 91.58% of the patients (Tao et al., 2021).

Polypharmacy can be a rational response for managing complex health problems in older adults (Wise, 2013). However, when the risk of adverse reactions caused by multiple drugs exceeds the benefits to patients, multidrug treatment becomes inappropriate and has been linked to numerous negative clinical outcomes, such as potentially inappropriate medications (PIMs), frailty, hospitalization, and even mortality (Wastesson et al., 2018). PIMs are defined as medication when their adverse risks exceed the health benefits compared to alternative therapies and when they should be avoided or replaced by more tolerable alternative drugs (Corsonello et al., 2009). PIM use in the elderly is becoming an important public health challenge and has been associated with a range of adverse events, including adverse drug reactions, falls, fractures, cognitive dysfunction, re-hospitalization, and death, along with increased health expenditure (Hyttinen et al., 2016; Wang et al., 2019). Therefore, monitoring and minimizing the prevalence of PIM use among older adults has become increasingly important. Up to date, several tools have been developed to identify PIM use in the elderly, among which the Beers criteria (2019), the Screening Tool of Older Persons’ Prescriptions (STOPP), and the Screening Tool to Alert to Right Treatment (START) criteria are the most popular guidelines (American Geriatrics Society Beers Criteria^®^ Update Expert Panel, 2019; Gallagher et al., 2008; O’Mahony et al., 2015). The Beers and STOPP criteria aim to identify PIMs, whereas the START criteria aim to identify potential prescription omissions (PPOs). In China, the criteria for potentially inappropriate medication for older adults (Chinese criteria) including medication risk and medication risk under morbid state have also been widely used (Wang, 2017). In total, a sum of 13 categories 72 medications or medication classes were selected in medication risk part, for example, neurologic medication, psychotropic medication, antipyretic, analgesic and anti-inflammatory medication and cardiovascular medication. PIM in the elderly under morbid state contained 44 medications or medication classes under 27 kinds of morbid states.

High prevalence rates of PIM use vary in different healthcare settings, from 20.6% to 87.4% (Tommelein et al., 2015; Sheikh-Taha and Dimassi, 2017; Zhang et al., 2017; Buda et al., 2020; Fernández et al., 2021; Bai et al., 2022). This study aimed to investigate the prevalence of polypharmacy, PIM use, and the factors associated with PIM use in elderly people aged ≥65 admitted to the Geriatric Department of Shandong Provincial Hospital affiliated to Shandong First Medical University in order to monitor and minimize PIM use.

## 2 Methods

### 2.1 Study design and participants

This cross-sectional study was conducted in the Geriatric Department of Shandong Provincial Hospital affiliated to Shandong First Medical University. Shandong Provincial Hospital is a government-run tertiary teaching hospital established in 1897 with 11 sub-professional departments in department of Geriatrics, including the cardiovascular, neurology, respiratory, digestive, endocrine, and hematology-oncology departments. A total of 276 in-patients aged 65 or older who received at least one medication during hospitalization between January 2020 to June 2020 were included in our study. This study was approved by the Medical Ethics Committee of Shandong Provincial Hospital affiliated with Shandong First Medical University (SWYX:NO. 2021–222).

### 2.2 Data collection

The electronic medical record systems (EMRs) of the patients were retrieved from the hospital information management system of Shandong Provincial Hospital affiliated to Shandong First Medical University. The retrieved data included sex, age, diagnosis, discharge medications (including oral medication, inhalers, patches, combination products), blood pressure, heart rate, body weight, and laboratory indicators (aspartate transaminase, alanine aminotransferase, total bilirubin, direct bilirubin, indirect bilirubin, albumin, blood urea nitrogen, creatinine, estimated glomerular filtration rate, white blood cell count, hemoglobin, platelet, prothrombin time, activated partial thromboplastin time, prothrombin time/international normalization ratio and D-dimer levels). Medications were coded using an anatomical therapeutic chemical classification system (W.H. Organization, 2003). International Classification of diseases (ICD) was used for identification of disease diagnoses (W.H. Organization, 2014), and disease diagnoses were derived from diagnostic information or disease conditions comprehensively recorded in the EMRs. Three authors independently extracted and analyzed the relevant data. Disagreements were resolved through discussions with rational drug use groups including clinicians, pharmacists, and network engineers.

### 2.3 Evaluation of PIM use

PIMs were evaluated based on the 2019 Beers criteria supported by the American Geriatric Society (American Geriatrics Society Beers Criteria^®^ Update Expert Panel, 2019), and five types of criteria were identified: 1) medications that should be avoided; 2) medications with drug-disease/syndrome interactions; 3) medications that should be used with caution; 4) medications with clinically important drug-drug interactions, and 5) medications that should be adjusted considering kidney function.

In addition, PIMs in older people were evaluated based on the Chinese and STOPP/START criteria version 2 in our study (Wang., 2017; Gallagher et al., 2008; O’Mahony et al., 2015).

Two authors manually identified the PIMs and a third author verified all items. All the authors discussed any discrepancies until a consensus was reached.

### 2.4 Statistical analysis

Numerical variables were examined for normal distribution and expressed as medians with interquartile ranges (IQR), while categorical data were expressed as numbers and proportions. The chi-square test was used to compare nominal categorical variables, and the Mann-Whitney *U* test was used to compare ordinal categorical variables. The Kappa test was used to evaluate the consistency of the four screening tools. A kappa>0.75 represented good to excellent agreement, 0.40<kappa<0.75 represented moderate agreement, and kappa<0.40 represented poor agreement. Spearman’s correlation was conducted to analyse correlation between different factors. Logistic regression analysis was conducted to identify independent factors associated with PIM use, including sex, age, number of diagnosed diseases, and number of prescribed medications. All *p* values were two-sided, and statistical significance was defined as *p* < 0.05.

Statistical analysis was performed with SPSS 26.0 software (IBM SPSS statistics for Windows, version 26.0, IBM Corp, Armonk, NY). Figures were drawn using the GraphPad Prism 8.0 software (GraphPad Software Inc., La Jolla, CA, United States).

## 3 Results

### 3.1 Patient characteristics

In total, 276 patients were enrolled in this study. Table 1 shows the characteristics of the study population.166 patients were male (60.14%), and 110 patients were female (39.86%). The median age was 72 years (IQR = 67–79), of which 168 patients (accounting for 60.87%) were aged 65–74 years, 80 patients (accounting for 28.99%) were aged 75–84 years, while 28 patients (accounting for10.14%) were 85 years old or more.

**TABLE 1:** Characteristics of the study population.

Variables	Groups		
Age (yrs) (n [%])	65–74	75–84	≥85
	168 (60.87%)	80 (28.99%)	28 (10.14%)
Age (yrs) (median [IQR])	72 years (IQR = 67–79)		
Gender (n [%])	Male		Female
	166 (60.14%)		110 (39.86%)
No. Prescribed medication (n [%])	1–4	5–9	≥10
	24 (8.70%)	132 (47.82%)	120 (43.48%)
No. Prescribed medication (median [IQR])	9 (IQR = 6.25–11)		
No. Diagnosed disease (n [%])	1–4	≥5	
	114 (41.31%)	162 (58.69%)	
No. Diagnosed disease (median [IQR])	5 (IQR = 3–7)		

The median number of comorbidities of the patients was five (IQR = 3–7). Overall, 40.31% (n = 114) of the patients had 1 to 4 comorbidities and 58.69% of patients (n = 162) had five or more comorbidities. The top five diseases were hypertension, coronary vascular disease, type 2 diabetes, cerebrovascular disease, and chronic obstructive pulmonary disease.

### 3.2 Polypharmacy

The median number of medications prescribed to patients was nine (IQR = 6.25–11), ranging from one to 28. Overall, 8.70% of patients took one to four medications, 47.82% took five to nine medications, and 43.48% took 10 or more medications (Table 1). In total, 91.30% of patients took five or more medications (which is defined as polypharmacy) and 43.48% of patients took 10 or more medications (which is defined as hyperpolypharmacy). Further analysis using Spearman’s correlation showed that the number of prescribed medications increased with age (rs = 0.144, *p* = 0.017).

### 3.3 PIM use based on different criteria

#### 3.3.1 PIM use based on beers criteria

The number of PIMs analyzed by the Beers criteria ranged between one and six.

**FIGURE 1:**
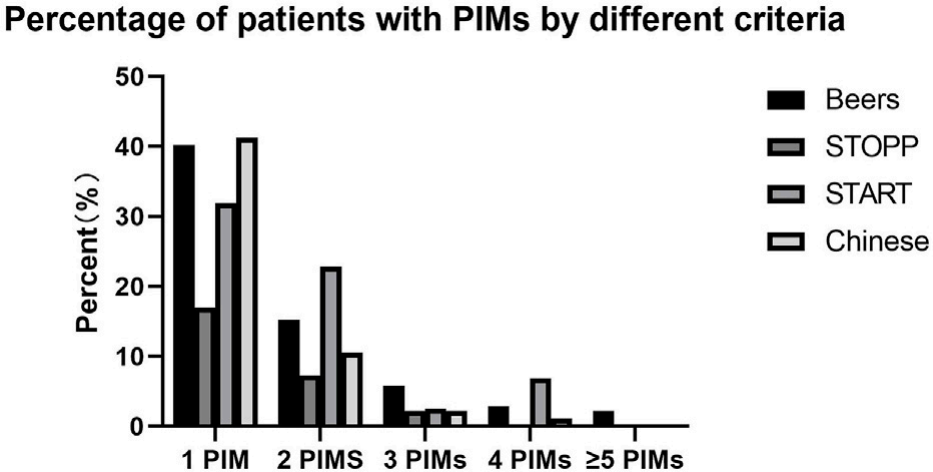
Percentage of patients with PIMs by different criteria.

One PIM was prescribed in 111 patients (40.22%), two PIMs in 42 patients (15.22%), three PIMs in 16 patients (5.80%), four PIMs in eight patients (2.90%), five PIMs in four patients (1.45%), and six PIMs in two patients (0.72%). PIMs were identified in 183 patients, accounting for 66.30% of the study population (Figure 1).

Among 276 elderly patients, 307 cases of PIMs were identified, including 206 cases (67.10%) of drug-related PIMs, 93 cases (30.29%) of drugs that should be used with caution, four cases (1.30%) of disease- or symptom-related PIMs, three cases (0.98%) of PIMs related to drug interaction, and one case (0.33%) of drugs that should be avoided or reduced due to renal insufficiency (Table 2).

**TABLE 2:** PIMs use based on Beers criteria (2019 version).

Beers criteria (2019 version)	Drugs	Suggestion	Cases (%)
drug related PIMs	proton pump inhibitor	avoid>8 weeks, unless high-risk	127 (41.37)
	benzenediazepine receptor agonist hypnotics: zopiclone, zopidem	avoid	10 (3.26)
	short acting and medium acting benzodiazepines: alprazolam and estazolam	avoid	30 (9.77)
	long acting benzodiazepines: clonazepam, diazepam	avoid	3 (0.98)
	glimepiride	avoid	10 (3.26)
	insulin (sliding dose)	avoid	1 (0.33)
	antipsychotic drugs: quetiapine, olanzapine	avoid	9 (2.93)
	oral noncyclooxygenase selective non steroidal anti-inflammatory drugs: diclofenac, ibuprofen	avoid long-term use unless other alternatives are ineffective	5 (1.63)
	indometacin	avoid	2 (0.65)
	amiodarone	avoid as a first-line treatment for atrial fibrillation	6 (1.95)
	digoxin as a first-line treatment for atrial fibrillation	avoid as a first-line treatment for atrial fibrillation	2 (0.65)
	anticholinergic drug: chlorphenamine	avoid	1 (0.33)
disease or symptom related PIMs	alzheimer’s disease combined with antipsychotics	avoid	2 (0.65)
	chronic nephropathy grade IV and above with NSAID	avoid	1 (0.33)
	heart failure with diltiazem	avoid or use with caution	1 (0.33)
drugs used with caution	diuretic	use with caution	75 (24.43)
	dabigatran, levashaban	use caution when treating VTE or atrial fibrillation in patients≥75 years old	3 (0.98)
	antipsychotic drugs (quetiapine, olanzapine)	use with caution	9 (2.93)
	oxcarbazepine	use with caution	1 (0.33)
	SSRI	use with caution	5 (1.63)
PIMs related to drug interaction	antidepressants (TCAs, SSRIs and SNRIs); antipsychotic drugs; antiepileptic; benzenediazepines; benzenediazepine receptor agonists hypnotics; opioids	avoid the combination of three or more CNS drugs	3 (0.98)
Drugs that should be avoided or reduced based on renal function	Creatinine clearance rate<30: spironolactone	avoid	1 (0.33)

The highest three PIM incidence rates involved proton pump inhibitors (127 cases, accounting for 41.37%), diuretics (75 cases, accounting for 24.43%), and benzodiazepines (alprazolam and estazolam; 30 cases, accounting for 9.77%), as shown in Table 2.

#### 3.3.2 PIM use based on the Chinese criteria

The number of PIMs analyzed using the Chinese criteria ranged from one to four. One PIM was observed in 114 patients (41.30%), two PIMs in 29 patients (10.51%), three PIMs in six patients (2.17%), and four PIMs in three patients (1.09%). PIMs were identified in 152 patients, accounting for 55.07% of the study population (Figure 1).

Among 276 elderly patients, 202 cases of PIMs from 152 patients were identified, including 30 high-risk and 165 low-risk cases. In the disease state, five cases were PIM grade A and two cases were PIM grade B. The highest three PIM incidence rates involved clopidogrel (98 cases, accounting for 48.51%), insulin (31 cases, accounting for 15.35%), and alprazolam (26 cases, accounting for 12.87%) (Table 3).

**TABLE 3:** PIMs use based on Chinese Criteria.

	Risk	Drugs	Cases (/%)
grade A warning	high-risk	alprazolam	26 (9.42)
	low-risk	eszolam	3 (1.09)
		negomelin	5 (1.81)
		zolpidem	3 (1.09)
		olanzapine	7 (3.47)
		quetiapine	1 (0.5)
		diclofenac	2 (0.99)
		ibuprofen	2 (0.99)
		digoxin	2 (0.99)
		amiodarone	6 (2.97)
		chlorphenamine	1 (0.5)
		insulin	31 (15.35)
		clopidogrel	98 (48.51)
		spironolactone	2 (0.99)
		theophylline	2 (0.99)
grade B warning	high-risk	nitrazepam	1 (0.5)
		indometacin	2 (0.99)
		aminoglycosides	1 (0.5)
disease or symptom related PIMs	grade A	diabetes combined with glucocorticoid	2 (0.99)
	grade A	prostatic hyperplasia combined with anticholinergics	1 (0.5)
	grade A	chronic gastric ulcer with glucocorticoid	1 (0.5)
	grade A	heart failure combined with diltiazem	1 (0.5)
	grade B	hypertension combined with reserpine	2 (0.99)

#### 3.3.3 PIM use based on the STOPP criteria

The number of PIMs analyzed using the STOPP criteria ranged from one to three. One PIM was observed in 47 patients (17.03%), two PIMs in 20 patients (7.25%), and three PIMs in six patients (2.17%). PIMs were identified in 73 patients, accounting for 26.45% of the study population (Figure 1).

Based on the STOPP standard, 105 PIMs involving 16 items were examined. The highest frequency of PIMs was “the elderly use drugs that may increase the risk of falls, such as benzodiazepine-alprazolam, clonazepam, midazolam, estazolam, diazepam” with a total of 36 cases (accounting for 34.29%), followed by “no monitoring of blood potassium when the aldosterone antagonist (spironolactone) was used in combination with other potassium retaining drugs (ARBs)" with a total of 17 cases (accounting for 16.19%). “Loop diuretics as a first-line drug for hypertension” was found in 16 cases (accounting for 15.24%) (Table 4).

**TABLE 4:** PIMs use based on STOPP criteria.

	Drugs	Cases (%)
Drugs that adversely affect fallers	Antipsychotic drugs: quetiapine, olanzapine	7 (6.67)
	Hypnotic Z-drugs: zopiclone, zopidem	10 (9.52)
	Benzodiazepines: alprazolam, estazolam, diazepam, clonazepam, midazolam	36 (34.29)
Cardiovascular system	β Receptor blockers combined with diltiazem (risk of heart block)	1 (0.95)
	Blood potassium was not monitored when aldosterone antagonist used in combination with other potassium retaining drugs (risk of hyperkalemia)	17 (16.19)
	Loop diuretics as a first-line drug for hypertension	16 (15.24)
	Amiodarone as a first-line drug for supraventricular arrhythmia	2 (1.9)
	Digoxin in patients with heart failure with normal ventricular systolic function	1 (0.95)
Antiplatelet and anticoagulant drugs	Aspirin plus clopidogrel as a secondary prevention of stroke	2 (1.9)
	Aspirin combined with direct thrombin inhibitor and factor Xa inhibitor for chronic atrial fibrillation	4 (3.81)
Urogenital system	Metformin when eGFR < 30 ml·min^−1^·1.73·m^−2^ (may cause lactic acid poisoning)	1 (0.95)
Gastrointestinal system	Oral iron more than 200 mg/d (no evidence that higher doses can increase iron absorption)	2 (1.9)
Duplicate drug classes	PPI	1 (0.95)
Central nervous system and psychotropic drugs	Antipsychotic drugs for the mental behavior of dementia (increasing the risk of stroke)	2 (1.9)
	Antipsychotic drugs (except quetiapine and clozapine) for patients with Parkinson’s disease	1 (0.95)
Musculoskeletal system	Glucocorticoid is used for osteoarthritis (risk of adverse reaction of systemic glucocorticoid application)	2 (1.9)

#### 3.3.4 PIM use based on START criteria

The number of PIMs analyzed using the START criteria ranged from one to four. One PIM was prescribed in 88 patients (31.88%), two PIMs in 63 patients (22.83%), three PIMs in seven patients (2.54%), and four PIMs in 19 patients (6.88%). PIMs were identified in 177 patients, accounting for 64.13% of the study population (Figure 1).

According to the START criteria, 311 patients were screened for missing prescriptions. Missed use of ACEI with systolic heart failure and/or coronary artery disease was found in 162 cases (accounting for 52.09%), while missed use of *β*-receptor blockers in patients with ischemic heart disease was found in 74 cases (accounting for 23.79%).

### 3.4 Factors associated with PIM use

Based on the four different criteria, the PIM group demonstrated significant differences in prescribed medications, age, and comorbidities (Table 5), although there was poor agreement among the four criteria analyzed by Kappa Statistic (kappa<0.40).

**TABLE 5:** Characteristics of 276 elderly participants identified based on four criteria.

Criteria	Viables	PIMs	Non-PIMs	*p*-Value
Beers		183	93	
	Age (yrs) (median [IQR])	72 (67–79)	72 (67.5–79.5)	*p* = 0.8660
	No. Prescribed medication (median [IQR])	10 (7–12)	7 (5–9)	*p* < 0.0001
	No.diagnosed disease (median [IQR])	5 (3–7)	6 (4–7)	*p* = 0.0523
Chinese		152	124	
	Age (yrs) (median [IQR]	73 (68–79)	71.5 (67–77)	*p* = 0.2212
	No. Prescribed medication (median [IQR])	10 (7–12)	8 (5–10)	*p* < 0.0001
	No.diagnosed disease (median [IQR])	5 (3–6)	6 (3–7)	*p* = 0.0671
STOPP		73	203	
	Age (yrs) (median [IQR]	75 (69–81)	71 (67–77)	*p* = 0.0032
	No. Prescribed medication (median [IQR])	11 (8–14)	8 (6–11)	*p* < 0.0001
	No.diagnosed disease (median [IQR])	6 (4–7)	5 (3–6)	*p =* 0.0037
START		177	99	
	Age (yrs) (median [IQR]	73 (67–79)	71 (67–78)	*p = 0.4784*
	No. Prescribed medication (median [IQR])	9 (7–12)	7 (5–11)	*p = 0.0004*
	No.diagnosed disease (median [IQR])	5 (3–6)	6 (4–6)	*p = 0.0001*

The prescribed medications in the PIM group were significantly higher than those in the non-PIM group based on all four criteria. The incidence of PIMs increased with an increase in the number of prescribed medications. Based on the 2019 Beers criteria, the incidence of PIMs was 20.83% for prescriptions of 1-4 medications, it increased to 60.61% for prescriptions of 5-9 medications, and it reached 81.67% for prescriptions of 10 or more medications (Table 6). Binary logistic regression analysis revealed that the use of PIMs was significantly associated with polypharmacy (Table 7). Analyzed by the Beers criteria, the risk of PIMs increased by 1.24 fold for each additional prescribed medication. Compared to prescriptions of 1-4 medications, the risk of PIM use for prescriptions of 5-9 medications soared to 5.161 (95% confidence interval [CI]: 1.791–14.866), and it increased to 15.858 (95% confidence interval [CI]: 5.304–47.535) for prescriptions of 10 or more medications. Similar results were obtained based on other criteria.

**TABLE 6:** Demographic and clinical characteristics of 276 patients and prevalence of PIMs.

Criteria	Viable	Overall (n = 276)	PIMs (n = 183)	Non-PIMs (n = 93)	*p*-Value
Gender (n [%])					
Beers	Male	166	108 (65.06)	58 (34.94)	0.5911
	Female	110	75 (68.18)	35 (31.82)	
STOPP	Male	166	39 (23.49)	127 (76.51)	0.1715
	Female	110	34 (31.91)	76 (69.09)	
Chinese	Male	166	97 (58.43)	69 (41.57)	0.1679
	Female	110	55 (50.00)	55 (50.00)	
START	Male	166	107 (64.46)	59 (35.54)	0.8892
	Female	110	70 (63.64)	40 (36.36)	
Age (yrs) (n [%])					
Beers	65–74	168	113 (67.26)	55 (59.14)	0.5565
	75–84	80	54 (67.50)	26 (27.96)	
	≥85	28	16 (57.14)	12 (12.90)	
Chinese	65–74	168	88 (52.38)	80 (47.62)	0.5248
	75–84	80	47 (58.75)	33 (41.25)	
	≥85	28	17 (60.71)	11 (39.29)	
STOPP	65–74	168	36 (21.69)	132 (79.52)	0.0056
	75–84	80	23 (28.75)	57 (71.25)	
	≥85	28	14 (50.00)	14 (50.00)	
START	65–74	168	105 (62.50)	63 (37.50)	0.5832
	75–84	80	55 (68.75)	25 (31.25)	
	≥85	28	17 (60.71)	11 (39.29)	
No.prescribed medication (n [%])					
Beers	1–4	24	5 (20.83)	19 (79.17)	<0.0001
	5–9	132	80 (60.61)	52 (39.39)	
	≥10	120	98 (81.67)	22 (18.33)	
Chinese	1–4	24	3 (12.50)	21 (87.50)	<0.0001
	5–9	132	68 (51.52)	64 (48.48)	
	≥10	120	81 (67.50)	39 (32.50)	
STOPP	1–4	24	3 (12.50)	21 (87.50)	0.0004
	5–9	132	24 (18.18)	108 (81.82)	
	≥10	120	46 (38.33)	74 (61.67)	
START	1–4	24	8 (33.33)	16 (66.67)	0.0022
	5–9	132	84 (63.64)	48 (36.36)	
	≥10	120	85 (65.38)	35 (29.17)	
No.diagnosed disease (n [%])					
Beers	1–4	114	84 (73.68)	30 (26.32)	0.0296
	≥5	162	99 (61.11)	63 (38.89)	
Chinese	1–4	114	69 (60.53)	45 (39.47)	0.1265
	≥5	162	83 (51.23)	79 (48.77)	
STOPP	1–4	114	22 (19.30)	92 (80.70)	0.0239
	≥5	162	51 (31.48)	111 (68.52)	
START	1–4	114	83 (72.81)	31 (27.19)	0.0117
	≥5	162	94 (58.02)	68 (41.98)	

**TABLE 7:** Multivariable regression of risk factors associated with PIMs use.

	Beers			Chinese			STOPP			START		
Viables	OR	95% CI	*p*-Value	OR	95% CI	*p*-Value	OR	95% CI	*p*-Value	OR	95% CI	*p*-Value
Gender												
Male	1 (ref)	_	_	1 (ref)	_	_	1 (ref)	_	_	1 (ref)	_	_
Female	0.905	0.516–1.587	0.727	0.661	0.393–1.112	0.119	1.780	0.991–3.197	0.054	0.894	0.529–1.514	0.678
Age			0.775			0.398			0.020			0.551
65–74	1 (ref)	_	_	1 (ref)	_	_	1 (ref)	_	_	1 (ref)	_	_
75–84	0.961	0.517–1.785	0.899	1.294	0.729–2.299	0.378	1.320	0.695–2.508	0.396	1.387	0.769–2.503	0.277
≥85	0.719	0.290–1.781	0.719	1.747	0.701–4.354	0.231	3.533	1.457–8.563	0.005	1.169	0.490–2.790	0.725
No.prescribed medication			0.000			0.000			0.001			0.008
1–4	1 (ref)	_	_	1 (ref)	_	_	1 (ref)	_	_	1 (ref)	_	_
5–9	5.161	1.791–14.866	0.002	7.193	2.018–25.636	0.002	2.261	0.584–8.760	0.238	3.068	1.208–7.793	0.018
≥10	15.878	5.304–47.535	0.000	14.193	3.953–50.958	0.000	6.132	1.612–23.324	0.008	4.447	1.730–11.432	0.002
No.diagnosed disease												
1–4	1 (ref)	_	_	1 (ref)	_	_	1 (ref)	_	_	1 (ref)	_	_
≥5	0.609	0.343–1.080	0.090	0.651	0.384–1.104	0.111	1.936	1.044–3.587	0.036	0.523	0.303–0.902	0.020

The age in the PIM group (median [IQR]:75 [69–81]) was higher than that in the non-PIM group (median [IQR]:71 [67–77]) based on the STOPP criteria; however, there were no significant differences between the two groups based on the other three criteria. The incidence of PIMs was 21.43% in participants aged 65–74 years old, it increased to 28.75% in participants aged 75–84 years old, and it reached the highest value of 50% in participants aged 85 years and more. A significant difference was found between participants aged 65–74 years and those aged 85 and more and between participants aged 75–84 years and those aged 85 years and more using the Chi-square test (*p* < 0.05). Binary logistic regression analysis revealed that the use of PIMs was significantly associated with age. Compared with participants aged 65–74 years, the risk of PIM use in participants aged 75–84 years soared to 1.320 (95% confidence interval [CI]: 0.695–2.508, *p* > 0.05), and it increased to 6.132 (95% confidence interval [CI]: 1.612–23.324, *p* = 0.008) in participants aged 85 years and more.

Significant differences in comorbidities were also found between the PIM and non-PIM groups, based on the STOPP and START criteria; however, the results were inconsistent. The number of diagnosed diseases in the PIM group was higher than that in the non-PIM group based on the STOPP criteria and lower than that in the non-PIM group based on the START criteria.

No significant association was found between gender and PIM use in our study.

## 4 Discussion

In the context of global population aging, age-associated multimorbidity and polypharmacy are widespread among the elderly and pose challenges to health and social care systems. In our study, 98.91% of elderly had multimorbidities, polypharmacy was found in 91.30% of older people aged 65 years or more, and the number of prescribed medications increased with age. Hyperpolypharmacy was also common in up to 43.48% of the older individuals. Our results are consistent with those of Tao et al. (Tao et al., 2021), but higher than those of previous reports (Lu et al., 2015; Bhatt et al., 2019; Bhagavathula et al., 2021; Chen et al., 2021). A systematic review and meta-analysis reported that the pooled prevalence of polypharmacy was 49%, and hyperpolypharmacy was 31% in older adults in India (Bhagavathula et al., 2021). The prevalence rates of polypharmacy and hyperpolypharmacy were 45.8% and 13.5%, respectively, in another study from two teaching hospitals in Southern India (Bhatt et al., 2019). A study from Taiwan, which used Taiwan’s Longitudinal Health Insurance Database to assess drug use for older adults, showed that polypharmacy was present in 28.2% of cases (Lu et al., 2015). The prevalence of polypharmacy was reported to account for 50.14% among older patients in a Chinese study (Chen et al., 2021). The inconsistency between different studies may be due to differences in the populations and areas; the subjects of our research were hospitalized elderly patients, whereas other reports were from outpatients or the Health Insurance Database.

Polypharmacy and hyperpolypharmacy are proxy indicators of PIM use in older populations and can lead to adverse clinical outcomes. Several tools are used to identify PIM use. Here, we used four different criteria, including the Beers, Chinese, STOPP, and START criteria, to analyze the incidence of PIM use. Similar results were found: PIMs were identified in 66.30% (183/276) of the study population based on the Beers criteria, 64.13% (177/276) based on the START criteria, and 55.07% (152/276) based on the Chinese criteria, whereas PIMs were identified only in 26.45% (73/276) of the elderly based on the STOPP criteria. The prevalence of PIMs varied widely among studies. Our study is consistent with some previous reports (Zhang et al., 2017; Buda et al., 2020; Bai et al., 2022), but inconsistent with others (Tommelein et al., 2015; Sheikh-Taha and Dimassi, 2017; Fernández et al., 2021). A total of 53.5% patients had at least one PIM identified by the Beers criteria (2015) reported by Zhang X et al. at Peking University First Hospital (Zhang et al., 2017). A total of 62.1% of patients had PIM use detected by the Chinese criteria, and 53.9% patients had PIM use detected by the 2019 Beers criteria in another study by Bai et al. at the Beijing Tongren Hospital (Bai et al., 2022). In the study by Bai et al., a low incidence of 20.6% was also found using the STOPP criteria. In addition, 25.80% patients had at least one PIM in Romania according to the STOPP criteria, though the prevalence of PPOs according to the START criteria was higher (41.72%) (Buda et al., 2020). The prevalence of PIMs was as high as 75% in community-dwelling older persons from five different cultural and social contexts: Kingston (Ontario, Canada), Saint-Hyacinthe (Quebec, Canada), Tirana (Albany), Manizales (Colombia), and Natal (Brazil) (Fernández et al., 2021). In a retrospective study conducted in a tertiary care center in the United States of America, PIM use reached 87.4% by the 2015 Beers criteria (Sheikh-Taha and Dimassi, 2017). The incidence of PIMs in our study was lower than that in a study conducted in Europe, with 22.6% PIM use in Europe (Tommelein et al., 2015). Many factors may lead to these differences, such as demographic differences, patient characteristics, differences in disease severity, the availability of clinical pharmacists or rational drug-use software, different criteria, or different versions of the same criteria.

The top three PIM incidence rates analyzed using the Beers criteria (2019) were those for proton pump inhibitors (PPI), diuretics, and benzodiazepines. 127 patients (accounting for 46.01%) had PIMs with proton pump inhibitors which to be avoided>8 weeks unless high-risk, accounting for 41.37% of all PIMs. Our results are consistent with those of previous studies. PPIs were reported as the most common PIMs used based on the Beers criteria in our study and in others (Zhang et al., 2017; Ma et al., 2018; Bhatt et al., 2019; Tian et al., 2021). The top three PIM incidence rates analyzed according to the Chinese criteria were those for clopidogrel, insulin, and alprazolam. 98 patients (accounting for 35.51%) had PIMs with clopidogrel accounting for 48.51% of all PIMs based on Chinese criteria. Consistent with previous study, clopidogrel is a common PIM used in most studies analyzed using the Chinese criteria (Ma et al., 2018; Tian et al., 2021). Benzodiazepines were the most frequently detected PIMs according to the STOPP criteria, 36 patients (accounting for 13.04%) had PIMs with benzodiazepines accounting for 32.29% of all PIMs. Although the consistency among the three criteria for PIM screening was poor, benzodiazepines were the most common PIMs detected by the Beers, Chinese, and STOPP criteria in our study and others (Parekh et al., 2019; Monteiro et al., 2020; Bai et al., 2022). In most reports, PPOs are commonly associated with the cardiovascular system. Missed use of ACEI with systolic heart failure and/or coronary artery disease was found as the most common PPO analyzed using the START criteria in our study, 162 patients (accounting for 58.70%) had PIMs with missing use of ACEI with systolic heart failure and/or coronary artery disease accounting for 52.09% of all PIMs, while the absence of antiplatelet therapy was reported as a highly ranked PPO in previous reports (Monteiro et al., 2020; Stojanović et al., 2020).

To help doctors and pharmacists identify high-risk patients, it is important to explore the risk factors that influence the incidence of PIMs. In our study, we found that the number of prescribed medications was the strongest independent predictor of PIMs, according to all four criteria. Consistent with our findings, several studies have reported a strong association between polypharmacy and PIM use (Ma et al., 2018; Fernández et al., 2021; Tao et al., 2021; Bai et al., 2022). The median number of medications prescribed to the participants was nine (IQR = 6.25–11), ranging from one to 28. Polypharmacy was very common in the elderly, and reached as high as 91.30%, whereas hyperpolypharmacy reached up to 43.48%. Compared to prescriptions of 1-4 medications, the risk of PIM use for prescriptions of 5-9 medications and 10 or more medications increased by 5.161 and 15.858 fold. These results indicate that reasonable reduction of prescriptions by clinical physicians and pharmacists without affecting efficacy may be a meaningful strategy for lowering the incidence of PIMs. Medication reviews and interventions have proven to be effective strategies for reducing polypharmacy and PIM use in the elderly (Ibrahim et al., 2021; Rodrigues et al., 2021; Stuhec et al., 2021; Stuhec and Zorjan, 2022). In a study of Stuhec et al., 246 patients receiving 3,294 medications were included, the clinical pharmacists proposed 374 interventions. Accepting clinical pharmacist recommendations reduced the total number of medications, the total number of prescribed PIMs, the number of potential drug-drug interactions which should be avoided (pXDDIs). Similarly, in another multicentric study including 243 patients with cardiovascular diseases, interventions of clinical pharmacists significantly improved the quality of pharmacotherapy prescribing by reducing the total number of medications and pXDDIs and led to better hypertension treatment guidelines adherence. In a systematic review including 47 articles involving 52–124,802 patients, various types of interventions were analyzed, and it was found that medication review was the most successful intervention in hospitals (Rodrigues et al., 2021). In another systematic review, the included studies suggested that deprescribing could be safe, feasible, well tolerated, and could lead to important benefits; the authors believed that deprescribing should focus on frailty status in high-risk populations (Ibrahim et al., 2021).

Age was another risk factor for PIMs based on the STOPP criteria in our study. The incidence of PIMs increased with age, from 21.43% to 50%. Binary logistic regression analysis revealed that the use of PIMs was significantly associated with age. Compared to participants aged 65–74 years, the risk of PIM use for participants aged 75–84 years and 85 years and older soared by 1.320 and 6.132 fold. A previous study identified older age as an independent factor associated with PIM use (Tao et al., 2021). These results indicate that more attention should be paid to the use of multiple medications in older adults. However, our results were only found using the STOPP criteria, and further study is required with a larger study population.

Our study has some limitations. First, it was a cross-sectional, observational, and single-center study. Second, our sample size is small and only inpatients aged 65 or more yeasrs old were included. In future studies, more elderly individuals, including those from community-dwelling and nursing homes, should be included. Third, no clinical outcoms were included, and the comparison results obtained by deprescribing the prescription with the guidance of clinical physicians and pharmacists should be included in further studies.

## 5 Conclusion

Similar results of a high prevalence of polypharmacy and PIM use in the elderly were observed based on all four criteria. Polypharmacy was identified as the strongest, independent factor associated with PIM use when analyzed by all four criteria. Older age was found to be an independent factor associated with PIM use according to the STOPP criteria only. A significant association between the extent of polypharmacy and the risk of PIM use was also found, indicating that reasonable reducing the number of drugs with the guidance of clinical physicians and pharmacists could be a meaningful strategy for lowering the incidence of PIMs.

## Data Availability

The original contributions presented in the study are included in the article/Supplementary material, further inquiries can be directed to the corresponding author.
